# Childhood obesity inequality in northeast China: joint effect of social economic status and school neighborhood environment

**DOI:** 10.1186/s12889-023-15194-w

**Published:** 2023-02-13

**Authors:** Yang Liu, Angela Cristina Bizzotto Trude, Shenzhi Song, Nan Jiang, Shihan Wang, Joel Gittelsohn, Deliang Wen

**Affiliations:** 1 Institute of Health Science, China Medical University, Shenyang, China; 2 Department of Pediatrics, Growth and Nutrition Division, University of Maryland School of Medicine, Baltimore, United States; 3 Institute of International Medical Education, China Medical University, Shenyang, China; 4 School of public health, China Medical University, Shenyang, China; 5 Department of International Health, Johns Hopkins Bloomberg School of Public Health, Human Nutrition Center, Baltimore, United States

**Keywords:** Childhood obesity, Social economic status, Neighborhood environment

## Abstract

**Background:**

Obesogenic environment is important in driving obesity epidemic. Children spend large amount of their time in schools. School neighborhood environment, as well as its interaction with socioeconomic status (SES) needs to be explored to provide evidence for children obesity prevention policies.

**Methods:**

Objective anthropometric measurement, a household structured questionnaire, and school geospatial analyses were carried out on 3670 children (aged 9–12 years) of 26 schools in northeast China. Interaction between SES inter-categorical intersectionality group and school neighborhood environment was tested for the effect on children’s body mass index z scores (z-BMI) and waist–hip ratio z scores (z-WHR), following formulation of SES inter-categorical intersectionality group based on household wealth, parental education, and parental occupation.

**Results:**

SES groups formed by household wealth, parental education and parental occupation was associated with z-BMI and z-WHR for girls. Those from moderate wealth & self-employed (M&S) families had the highest adjusted z-BMI and z-WHR among all SES groups. School neighborhood environment factors interacted with SES groups in association with WHR for girls. Number of school neighborhood supermarkets and residential sites were negatively associated with z-WHR for girls from M&S families (β= -0.45 (95%CI: -0.76, -0.15) for supermarkets; β= -0.01 (95%CI: -0.03, 0.00) for residential sites). Number of school neighborhood convenience stores and public transport stops were positively associated with z-WHR for girls from M&S families (β = 0.02 (95%CI: 0.00, 0.03) for convenience stores; β = 0.23 (95%CI: 0.15, 0.31) for public transport stops). While non-significant association was found for number of vegetable stores.

**Conclusion:**

Girls from moderate wealth & self-employed families may be the group susceptible to school neighborhood environment. Local policies targeted at improving the school neighborhood environment may be one avenue for reducing socioeconomic disparities in obesity especially for girls.

**Supplementary Information:**

The online version contains supplementary material available at 10.1186/s12889-023-15194-w.

## Introduction

An escalating global epidemic of obesity is taking over many parts of the world [1]. China has experienced a sharp increase in economic growth since 1978 and has become the country with the largest number of children with obesity in the world [2]. Obesogenic environment, which refers to an environment that promotes gaining weight and one that is not conducive to weight loss within the home or workplace, is important in driving obesity epidemic [3]. It depicts the obesogenic characteristics of the built environment items – buildings, roads, buses, homes, parks, recreational areas, greenways, shops and other business areas. Environment strategy that focusing on modifying the obesogenic environment at the community and policy level can impact people at large.

Schools are important settings for obesity prevention programs seeking to impact children at large [4]. Children’s health behaviors often vary considerably from school to school. Characteristics of the built environment in school neighborhood such as residential density, walkability, physical activity areas, and food outlets, are important factors contributing to the variation [5, 6]. School-based obesity prevention programs were reported fail to account for barriers students face to engaging in health behaviors outside of school or as they travel to and from school [7, 8]. However, programs changing obesogenic environment in school neighborhood were believed to be more useful in informing policy to support health behavior around schools than that operationalizing the neighborhood as the area surrounding the home [8].

On the other hand, individual characteristics should not be neglected when we try to address childhood obesity by changing obesogenic environment in school neighborhood, among which socioeconomic status (SES) is especially important. Inconsistent results were reported for association between childhood obesity and neighborhood environment by previous studies focusing on home neighborhoods and school neighborhoods [9–11]. It is possible that some of these inconsistencies are due to subgroup specific effects brought by moderation of SES including family income, parental education and parental occupation. In addition, different SES factors may have different and even opposite moderation effect. For example, income and wealth increases food access, while education and occupation may help people take healthy decisions where unhealthy food availability is ubiquitous [12, 13]. We could also see obesity-SES paradox that in developing countries obesity is a positively associated with the upper-SES class while this association reverses as economies develop [14, 15].

Instead of studying factors separately, intersectionality is often used as a theoretical framework that focuses on the ways that those at different SES strata are differently influenced by structural or interpersonal contexts [16, 17]. Intersectionality lens was used in the present study and a joint inequality of SES and school neighborhood environment was hypothesized to be existed on childhood obesity in northeast China. Two aims were included in the present study: firstly, to examine association of SES with children’s body mass index (BMI) and waist–hip ratio (WHR); Secondly, to examine association of school neighborhood environments with children’s BMI and WHR moderated by SES.

## Materials and methods

### Participants

We assessed a sample of Chinese children aged 9 through 12, which is a time of striking behavioral change related to the social and physical environment [18]. Our sample was drawn from 26 elementary schools in Shenyang, China. Shenyang is the largest city in northeast China by urban population and consists of 13 administrative districts, including 10 municipal districts of Shenyang proper, 1 count-level city, and 2 counties. According to the 2020 census, Shenyang’s total population had surpassed 9.0 million, with the urban population comprising 7.6 million of the total and children population at primary school age comprising 0.4 million of the total.

Two schools from each of the 13 administrative districts of Shenyang city were randomly selected. Then, one classroom from each of the fourth-, fifth-, and sixth-grade divisions of each school was randomly selected to be included in the study. All students from the selected classes and their parents were recruited as the sample of the present study, with their consent, and all participants had the option to withdraw from the study at any point (sixty students withdrew from the study). Finally, 3670 children were included into the study.

#### Institutional review board (IRB) approval statement and statement of patient consent

The study was approved by the China Medical University Ethics Committee (2017-055). Written informed consents were obtained from both parents and children before anthropometric measurement with ethics approved before the study.

#### Procedures

Survey and data collection was completed from May 2017 to June 2017, including anthropometric measurements, a household structured questionnaire, and objectively assessed neighborhood characteristics.

Anthropometric measurements—height, weight, waist circumference, and hip circumference—were carried out on physical examination day at each school by trained investigators using techniques prescribed by Lohman et al. [19] Weight was measured with subjects wearing no shoes and only light underwear. The measure was taken using a portable Tanita DC-430MA dual frequency body composition monitor (TANITA Corporation, Tokyo, Japan). Standing height was measured without shoes, by a Seca 213 portable stadiometer (Hamburg, Germany). Waist circumference (WC) (cm) was measured at the midpoint between the level of the xiphoid process and the top of the iliac crest, and hip circumference (HC) (cm) at the widest point around the buttocks.

A household structured questionnaire were handed out to each student three days prior to the physical examination, answered by one or both parents, and collected by the investigators at physical examination. Incomplete questionnaires or those with missing data were filtered by research personnel (SZS) and returned to parents for completion of any inadvertently missed portions, and handed back to school physicians.

Longitude and latitude of school addresses were collected by typing school names into Baidu map. The addresses were then confirmed when data collectors came to schools in person. The collected longitude and latitude were then typed into SuperMap GIS 9D for geospatial analyses on objectively assessed neighborhood characteristics.

#### Measures

*Body Mass Index (BMI) and Waist–hip ratio (WHR).* BMI was calculated as weight (kg) divided by height (m) squared. Age- and sex-specific Z-BMI scores were calculated using the WHO standard [20]. As there was no reference to normalize WHR, age- and sex-specific Z-WHR scores were calculated using the means and standard deviations of our study population to make the effect estimates comparable.

*Household wealth.* The procedure to generate the household wealth index had been previously described in detail [21]. The index was generated through a principal components analysis based on the following indicators: household income, food costs as a proportion of annual income, ratio of income to expenditure, self-reported evaluation of household income compared to the local average, income growth in the last three years, satisfaction of household income, number of private cars, number of computers, if the child has his/her own room, and number of family trips per year.

*Parental education.* Parental education consisted of father’s and mother’s highest education level. Education, according to the Chinese education system, was divided into eight categories: none, primary school, middle school, high school, technical secondary school, junior college, undergraduate, and postgraduate and above.

*Parental occupation.* Multiple choice questions were used in questionnaires to collect information parental occupation, which included: directors in government agencies and enterprises, professional or technical personnel, general staff, commercial/service workers, self-employed small business owner, non-agricultural workers, non-agricultural laboring farmers, farmers/laborers, other, and unemployed.

*Objectively assessed neighborhood characteristics.* Geospatial analyses were conducted using SuperMap GIS 9D. Participants’ school addresses were geocoded using the longitude and latitude coordinate system. Sites of interest with in 1 km circular buffer of each participant’s school were extracted from SuperMap GIS 9D and were counted in STATA 14.0. The following sites were included: food outlets (supermarkets, farmer markets, vegetable stores, convenience stores, confectionery stores, and fast food restaurants), residential sites, physical activity areas (parks, leisure squares, plazas, and amusement parks), public transport stops, and road intersections.

*Covariates.* Demographics information including age, gender, and puberty onset were collected in household structured questionnaire and used as covariates. Puberty onset was measured by responses reported by parents regarding first menses for girls and appearance of voice change, facial hair, or increase in size of Adam’s apple for boys.

#### Analysis

**Fig. 1:**
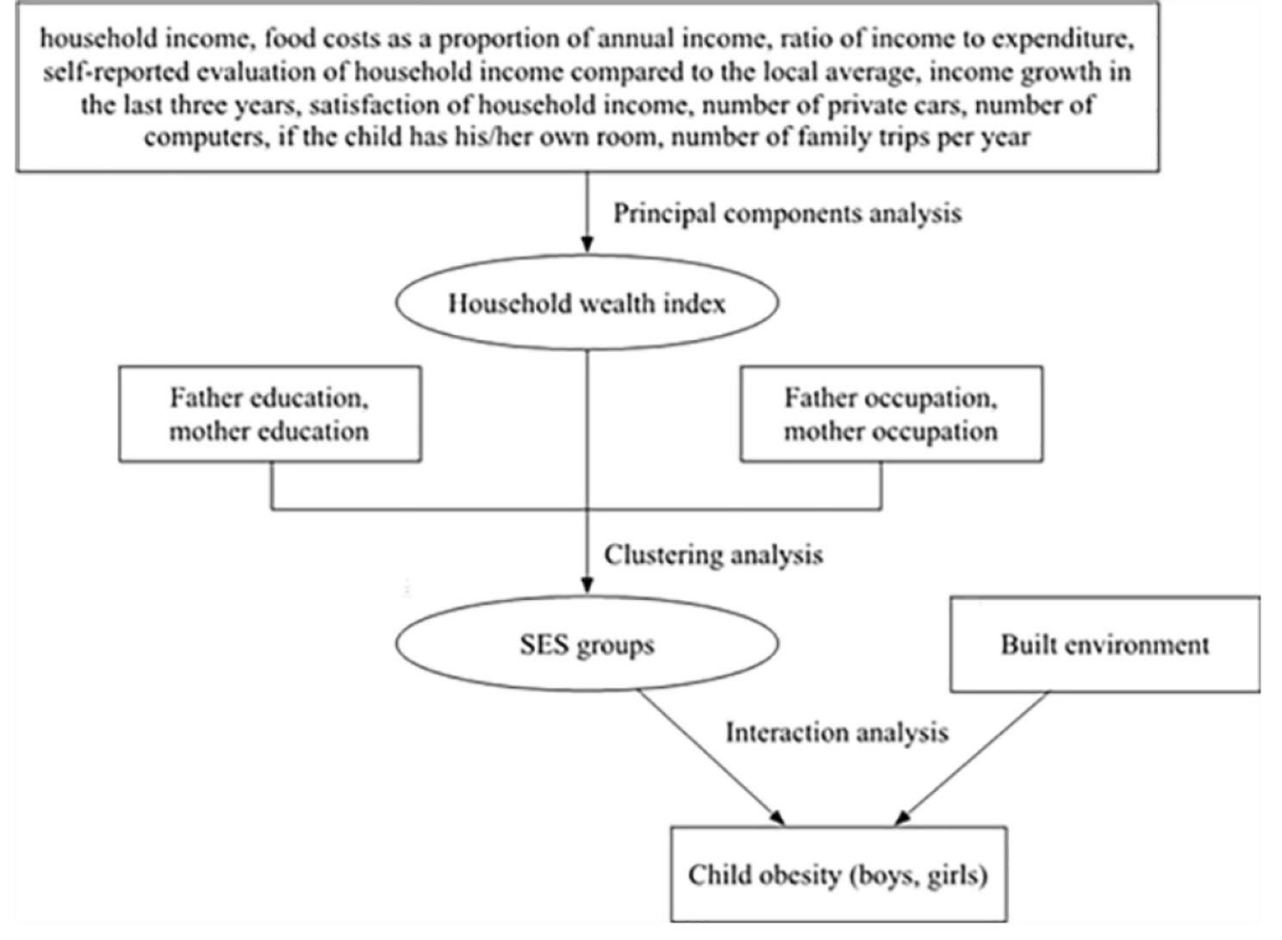
Diagram of analysis strategy to explore the joint inequality of various dimensions on childhood obesity

As the diagram of analysis strategy shown in Fig. 1, an inter-categorical intersectionality group of household wealth (based on 10 income and household wealth indicators), parental education, and parental occupation was generated to examine their effects on obesity risk in children. Then, an interaction between the SES inter-categorical intersectionality group and built environment was tested for its effect on obesity risk in children. Separate analyses for boys and for girls were conducted to take into consideration gender susceptibility for SES and built environment.

#### Step 1: Formulation of inter-categorical intersectionality group of household wealth, parental education, and parental occupation, and its relation to obesity risk in children

SES groups were generated based on how obesity was influenced by SES. According to the “Obesity Kuznets curve” and nutrition transition [22, 23], as income rises, people consume more calories, leading to an increase in obesity rates; as income continues to rise, personal health becomes a more valued asset, thus influencing the decrease in obesity levels. Therefore, this study focused on the heterogeneity of the middle and high SES groups [24]. In the present study, households were divided into three groups according to the latent household wealth score: the lowest 40% of household wealth index was classified as “poor”, the highest 20% as “rich”, and the remaining 40% as “middle class” [25]. For middle and rich classes, occupation and education were then used to strengthen the classification using the SPSS Two Step Clustering Component scalable analysis algorithm, which is capable of handling both continuous and categorical variables. In the first step, the records were pre-clustered into many small sub-clusters. Then, the sub-clusters from the pre-cluster step were clustered into the desired number of clusters.

Multilevel mixed-effects linear regression models (using STATA 14.0 MIXED commands) were fit for the relationship of composite SES groups with children’s BMI and with children’s WHR with accounting for clustering of participants within schools as random effects. As boys and girls differ in physical features related to obesity and were influenced by SES differently, analyses were performed separately for boys and for girls. Age and onset of puberty were adjusted as confounding variables.

#### Step 2: Interaction between the SES groups and built environment on children’s z-BMI and z-WHR

First, interaction terms between characteristics of the school neighborhood environment and SES were fitted in the mixed regression models. Separate mixed effects regression models were fit for each neighborhood characteristic. Age and onset of puberty were adjusted as confounding variables. Then marginal effects at representative values (MERs) were estimated and graphed to predict children’s z-BMI and z-WHR in different combinations of school neighborhood environment and SES.

## Results

### Generation of the composite SES groups

Six composite SES groups were identified, as shown in Table 1: poor families (*n* = 1476), moderate wealth & low parental education families (M&L, *n* = 632), moderate wealth & self-employed families (M&S, *n* = 359), moderate wealth & professional or technical worker families (M&P, *n* = 480), rich & self-employed families (R&S, *n* = 493), and rich & highly-educated families (R&H, *n* = 230).

**Table 1:** Composite social class groups based on household wealth, parental occupation, and parental education level

SES groups	Characteristics of the group	N (%)
Rich & Highly-educated families (R&H)	Father’s occupation: Directors in government agencies and enterprises, Professional or technical personnel, Commercial/ Service workers	230 (6.3)
	Mother’s occupation: Directors in government agencies and enterprises, Professional or technical personnel	
	Father’s education level: Junior college, Bachelor’s degree	
	Mother’s education level: Junior college, Bachelor’s degree	
Rich & Self-employed families (R&S)	Father’s occupation: Self-employed small business owner, Other	493 (13.4)
	Mother’s occupation: Self-employed small business owner, Other	
	Father’s education: Middle school, High school, Technical secondary school	
	Mother’s education: Middle school, High school, Technical secondary school	
Moderate wealth & Professional or technical worker families (M&P)	Father’s occupation: Professional or technical personnel, Directors in government agencies and enterprises, General staff	480 (13.1)
	Mother’s occupation: General staff, Professional or technical personnel, Commercial/ Service workers	
	Father’s education: High school, Technical secondary school, Junior college, Bachelor	
	Mother’s education: High school, Technical secondary school, Junior college, Bachelor	
Moderate wealth & Self-employed families (M&S)	Father’s occupation: Self-employed merchants	359 (9.8)
	Mother’s occupation: Self-employed merchants	
	Father’s education: Middle school, High school, Technical secondary school	
	Mother’s education: Middle school, High school, Technical secondary school	
Moderate wealth & Low parental education level families (M&L)	Father’s occupation: Other, Unemployed, Farmers/Laborers	632 (17.2)
	Mother’s occupation: Other, Unemployed, Farmers/Laborers	
	Father’s education: Primary school, Middle school	
	Mother’s education: Middle school, High school, Technical secondary school	
Poor families	The lowest 40% of household wealth	1476 (40.2)

### Characterization of sample

The distribution of the sample into socioeconomic groups and weight status was given in Table 2. Age and puberty were distributed differently for girls and for boys (*p* < 0.001). Mean BMI was 18.4 (SD 4.) among girls and 19.4 (SD 4.4) among boys; mean WHR was 0.8 (SD 0.1) among girls and 0.8 (SD 0.1) among boys.

**Table 2:** Distribution of demographic, socioeconomic groups, proportion of people with obesity, by sex

	Girls (n = 1799) n(%)	Boys (n = 1871) n(%)	*P* ^*1*^
Age (years)			< 0.001
< 9	2(0.1)	3(0.2)	
9	218(12.1)	162(8.7)	
10	603(33.5)	573(30.6)	
11	529(29.4)	619(33.1)	
12	428(23.8)	456(24.4)	
> 12	21(1.18)	58(3.1)	
Puberty onset (yes)	29(1.6)	170(9.1)	< 0.001
Household wealth^2^			0.23
Poor	360(20)	352(18.8)	
Middle	698(38.8)	776(41.5)	
Rich	741(41.2)	743(39.7)	
Father’s education level			0.08
None	4(0.2)	6(0.3)	
Primary school	160(8.9)	163(8.7)	
Middle school	792(44)	820(43.8)	
High school	354(19.7)	416(22.2)	
Technical secondary school	160(8.9)	144(7.7)	
Junior college	167(9.3)	200(10.7)	
Bachelor	144(8)	111(5.9)	
Master and above	18(1)	11(0.6)	
Mother’s education level^2^			0.27
None	11(0.6)	8(0.4)	
Primary school	146(8.1)	148(7.9)	
Middle school	826(45.9)	827(44.2)	
High school	320(17.8)	354(18.9)	
Technical secondary school	153(8.5)	168(9)	
Junior college	180(10)	229(12.2)	
Bachelor	150(8.3)	126(6.7)	
Master and above	13(0.7)	11(0.6)	
Father’s occupation^2^			0.16
Directors in government agencies and enterprises	144(8)	125(6.7)	
Professional or technical personnel	172(9.6)	217(11.6)	
General staff	136(7.6)	150(8)	
Commercial/Service workers	126(7)	118(6.3)	
Self-employed small business owners	329(18.3)	350(18.7)	
Non-agricultural workers	79(4.4)	99(5.3)	
Non-agricultural laboring farmers	56(3.1)	76(4.1)	
Farmers Laborers	205(11.4)	221(11.8)	
Other	435(24.2)	410(21.9)	
Unemployed	117(6.5)	105(5.6)	
Mother’s occupation			0.16
Directors in government agencies and enterprises	75(4.2)	75(4)	
Professional or technical personnel	113(6.3)	116(6.2)	
General staff	175(9.7)	180(9.6)	
Commercial/Service workers	221(12.3)	228(12.2)	
Self-employed small business owners	262(14.6)	279(14.9)	
Non-agricultural workers	49(2.7)	65(3.5)	
Non-agricultural workers	49(2.7)	65(3.5)	
Non-agricultural laboring farmers	43(2.4)	62(3.3)	
Farmers Laborers	182(10.1)	195(10.4)	
Other	407(22.6)	385(20.6)	
Unemployed	272(15.1)	286(15.3)	
BMI, mean ± SD ^3^	18.4 ± 4.0	19.4 ± 4.4	< 0.001
z-BMI, mean ± SD ^3^	-0.1 ± 0.9	0.1 ± 1.0	< 0.001
WHR, mean ± SD ^3^	0.8 ± 0.1	0.8 ± 0.1	< 0.001
z-WHR, mean ± SD ^3^	-0.1 ± 1.0	0.1 ± 0.9	< 0.001

^1^ Chi-square test was used. ^2^ Latent variables generated with PCA. ^3^ Two-sided t-test was used

### Association between SES group and z-BMI and z-WHR

Table 3 showed results based on multilevel mixed-effects linear regression for the relationship of composite SES groups with children’s BMI and with children’s WHR. Girls from M&S families had the highest adjusted z-BMI (0.28 z-BMI) and z-WHR (0.22 z-WHR). Compared with other groups, girls from M&S families had 0.18 higher z-BMI than those from poor families (95% CI: -0.36, 0.00, *p* = 0.05, Ref: M&S) and 0.25 higher z-BMI than those from M&L families (95% CI: -0.44, -0.05, p = 0.01, Ref: M&S). Girls from M&S families had higher z-WHR than all other families (coefficient ranged from − 0.33 to -0.23, *p* < 0.05 for all models, Ref: M&S).

**Table 3:** Adjusted mean of z-BMI and z-WHR in each subgroup and test for significance^1, 2^

	Girls				Boys			
	*Adjusted mean*	Coef	95%CI	*P*	*Adjusted mean*	Coef	95%CI	*P*
z-BMI								
Poor families	**0.10**	**-0.18**	**-0.36, 0.00**	0.05	0.02	-0.15	-0.35, 0.06	0.19
M&L families	**0.04**	**-0.25**	**-0.44, -0.05**	0.01	0.01	-0.16	-0.38, 0.07	0.18
M&S families	0.28	Ref^3^	Ref^3^	Ref^3^	0.03	-0.14	-0.38, 0.10	0.26
M&P families	0.13	-0.16	-0.36, 0.05	0.13	0.11	-0.06	-0.28, 0.17	0.66
R&S families	0.11	-0.18	-0.38, 0.03	0.09	0.01	-0.15	-0.39, 0.08	0.20
R&H families	0.14	-0.14	-0.38, 0.10	0.25	0.17	Ref^4^	Ref^4^	Ref^4^
**z-WHR**								
Poor families	**-0.01**	**-0.23**	**-0.41, -0.06**	0.01	0.00	0.22	0.00, 0.44	0.87
M&L families	**-0.01**	**-0.24**	**-0.43, -0.04**	0.02	-0.20	0.02	-0.23, 0.27	0.22
M&S families	0.22	Ref^5^	Ref^5^	Ref^5^	-0.10	0.12	-0.16, 0.40	0.40
M&P families	**-0.08**	**-0.30**	**-0.51, -0.09**	< 0.01	-0.10	0.12	-0.15, 0.39	0.76
R&S families	**-0.03**	**-0.26**	**-0.46, -0.05**	0.01	-0.22	Ref^6^	Ref^6^	Ref^6^
R&H families	**-0.11**	**-0.33**	**-0.57, -0.09**	0.01	-0.12	0.10	-0.25, 0.45	0.71

^1^ Multilevel mixed-effects linear regression models were fit for the relationship of composite SES groups with children’s BMI and with children’s WHR. All models were adjusted by age, and onset of puberty (initiation of menstrual periods for girls, appearance of voice change, facial hair, or increase in size of Adam’s apple for boys)

^2^ Results of four models were shown in the table: boys’ z-BMI, boys’ z-WHR, girls’ z-BMI and girls’ z-WHR

^3^ Girls from M&S families had the highest z-BMI and were selected as reference group

^4^ Boys from R&H families had the highest z-BMI and were selected as reference group

^5^ Girls from M&S families had the highest z-WHR and were selected as reference group

^6^ Boys from R&S families had the highest z-WHR and were selected as reference group

### Interaction between SES group and neighborhood environment on z-BMI and z-WHR

**Fig. 2:**
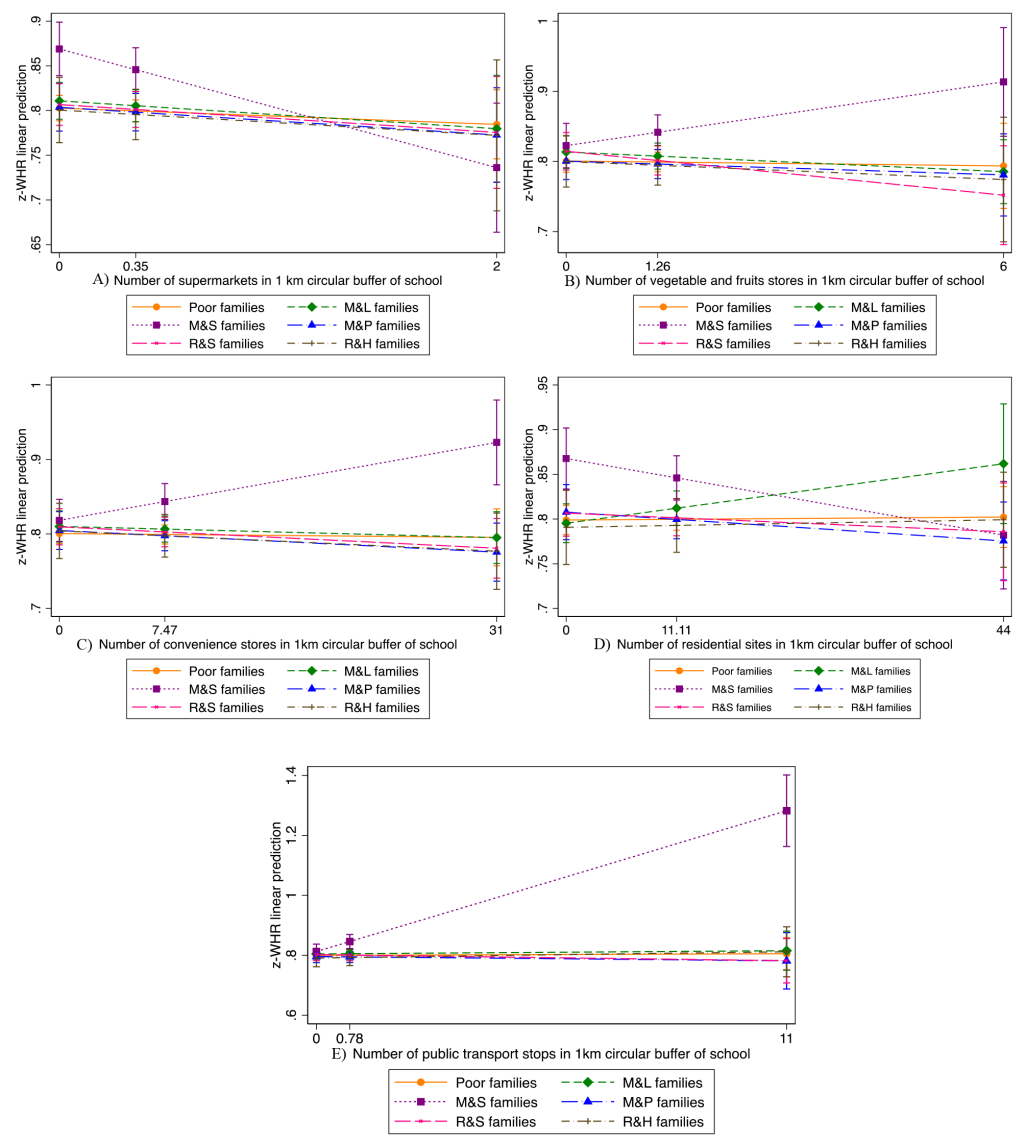
Interaction between SES groups and school-neighborhood environment on z-WHR for girls

Based on the results that association between SES group and z-BMI and z-WHR were only statistically significant for girls, further analysis was conducted on examining interaction between SES group and school neighborhood environment for girls. The interaction was not statistically significant for girls’ z-BMI (Tables S1 and S2) but was statistically significant for z-WHR. As shown in Table 4, school neighborhood environment factors interacting with SES included numbers of supermarkets, vegetables stores, convenience stores, residential sites, and public transport stops in 1 km circular buffer of schools (non-significant factors included farmer markets, confectionery stores, fast food stores, physical activity areas, and road intersections, as shown in Table S3). Figure 2 depicts the z-WHR linear prediction across different numbers of supermarkets, vegetables stores, convenience stores, residential sites, and public transport stops for different SES groups. For M&S families, each increase in number of school neighborhood supermarkets and residential sites were respectively related to 0.45 SD (95%CI: -0.76, -0.15) and 0.01 SD (95%CI: -0.03, 0.00) decrease in WHR for girls (Table 4; Fig. 2). Furthermore, each increase in number of school neighborhood convenience stores and public transport stops were respectively related to 0.02 SD (95%CI: 0.00, 0.03) and 0.23 SD (95%CI: 0.15, 0.31) increase in WHR for girls from M&S families. While the association between number of vegetable stores and WHR was non-significant for girls from M&S families (Coef; 95%CI: 0.09; -0.02, 0.20).

**Table 4:** Tests for SES-by-school neighborhood interactions on WHR for girls(Ref: M&S families)^1^

	Model 1			Model 2					Model 3		Model 4		Model 5		
	Coef	95%CI		Coef		95%CI			Coef	95%CI	Coef	95%CI	Coef		95%CI
SES group															
Poor families	-0.37	-0.60, -0.13		-0.11		-0.36, 0.15			-0.09	-0.32, 0.13	-0.49	-0.75, -0.22	-0.07		-0.28, 0.13
M&L families	-0.37	-0.62, -0.11		-0.06		-0.34, 0.21			-0.07	-0.33, 0.18	-0.51	-0.80, -0.23	-0.07		-0.30, 0.15
M&P families	-0.42	-0.71, -0.14		-0.14		-0.43, 0.15			-0.10	-0.38, 0.17	-0.42	-0.75, -0.10	-0.14		-0.37, 0.10
R&S families	-0.41	-0.68, -0.14		-0.02		-0.32, 0.27			-0.05	-0.32, 0.22	-0.43	-0.73, -0.13	-0.07		-0.31, 0.16
R&H families	-0.43	-0.76, -0.09		-0.17		-0.51, 0.17			-0.11	-0.45, 0.22	-0.54	-0.92, -0.16	-0.18		-0.46, 0.11
Number of supermarkets	**-0.45**	**-0.76, -0.15**													
Number of vegetable stores				0.09		-0.02, 0.20									
Number of convenience stores									**0.02**	**0.00, 0.03**					
Number of residential sites											**-0.01**	**-0.03, 0.00**			
Number of public transport stops													**0.23**		**0.15, 0.31**
Number of supermarkets* Poor families	0.31	-0.03, 0.66													
Number of supermarkets* M&L families	0.31	-0.08, 0.70													
Number of supermarkets* M&P families	0.33	-0.05, 0.71													
Number of supermarkets* R&S families	**0.40**	**0.00, 0.79**													
Number of supermarkets* R&H families	0.30	-0.17, 0.78													
Number of vegetable stores * Poor families				-0.10		-0.24, 0.04									
Number of vegetable stores * M&L families				-0.12		-0.26, 0.01									
Number of vegetable stores * M&P families				-0.12		-0.26, 0.02									
Number of vegetable stores * R&S families				**-0.18**		**-0.33, -0.03**									
Number of vegetable stores * R&H families				-0.11		-0.28, 0.06									
Number of convenience stores * Poor families									**-0.02**	**-0.04, 0**					
Number of convenience stores * M&L families									**-0.02**	**-0.04, 0**					
Number of convenience stores * M&P families									**-0.03**	**-0.05, -0.01**					
Number of convenience stores * R&S families									**-0.03**	**-0.05, -0.01**					
Number of convenience stores * R&H families									**-0.03**	**-0.05, 0**					
Number of residential sites * Poor families											**0.01**	**0.00, 0.03**			
Number of residential sites * M&L families											**0.02**	**0.01, 0.04**			
Number of residential sites * M&P families											0.01	-0.01, 0.02			
Number of residential sites * R&S families											0.01	-0.01, 0.03			
Number of residential sites * R&H families											**0.02**	**0.00, 0.03**			
Number of public transport stops * Poor families														**-0.23**	**-0.32, -0.13**
Number of public transport stops * M&L families														**-0.22**	**-0.32, -0.12**
Number of public transport stops * M&P families														**-0.24**	**-0.34, -0.13**
Number of public transport stops * R&S families														**-0.24**	**-0.33, -0.14**
Number of public transport stops * R&H families														**-0.21**	**-0.32, -0.11**
Constant	0.43		0.19, 0.67		0.12		-0.13, 0.38	0.11		-0.12, 0.35	0.48	-1.56, 2.51		0.06	-0.15, 0.28

^1^Adjusted for age, and onset of puberty (initiation of menstrual periods for girls, appearance of voice change, facial hair, or increase in size of Adam’s apple for boys)

Model 1: tested the effect modification between the number of **supermarkets** across SES groups in reference to M&S families. The model was specified as follows: SES group, number of supermarkets, number of supermarkets*SES group

Model 2: tested the effect modification between the number of **vegetable stores** across SES groups in reference to M&S families. The model was specified as follows: SES group, number of vegetable stores, number of vegetable stores*SES group

Model 3: tested the effect modification between the number of **convenient stores** across SES groups in reference to M&S families. The model was specified as follows: SES group, number of convenient stores, number of convenient stores*SES group

Model 4: tested the effect modification between the number of **residential sites** across SES groups in reference to M&S families. The model was specified as follows: SES group, number of residential sites, number of residential sites*SES group

Model 5: tested the effect modification between the number of **public transport stops** across SES groups in reference to M&S families. The model was specified as follows: SES group, number of public transport stops, number of public transport stops *SES group

## Discussion

To our best knowledge, the present study is one of the first to explore the joint inequality of household wealth, parental education, parental occupation, and school neighborhood environment on childhood obesity. The hypothesis on joint inequality of SES and school neighborhood environment was validated among girls. Firstly, girls from moderate wealth & self-employed families (M&S) had the highest adjusted z-BMI and z-WHR among all SES subgroups. Secondly, school neighborhood environment factors interacted with SES groups in association with WHR of girls. WHR of girls from M&S families was associated with number of supermarkets, residential sites, convenience stores, and public transport stops in 1 km circular buffer of school.

The result that the joint inequality for obesity risk was only found among girls was consistent with conclusions from previous studies indicating that females were more susceptible to social factors than males [26–29]. For example, one study conducted among American populations found that racial differences in obesity were only found among females [28]. Girls’ obesity was independently associated with parental education and employment status [30]. It’s reported that greater parental control had been linked to increased adiposity in females, but not males [29]. Relationships with parental activity were stronger for girls [29]. One study from the United States using a state level panel containing 4044 males and 4044 females from 1991 to 2010 found evidence of an Obesity Kuznets curve for white females but not for white males [31]. Interaction between wealth and education was found among women in a study using four datasets of women of reproductive age from the Egyptian Demographic and Health Surveys spanning two distinct time periods: 1992/95 (N = 11,097) and 2005/08 (N = 23,178) [32]; as well as among girls in a cross-sectional study containing 3670 children from northeast China [21].

Moderate wealth & self-employed families (M&S) were found to be the subgroup that had the highest obesity risk for girls. Our results showed that neither the poorest/lowest education group nor the richest/highest education group had the highest obesity risk. This result was consistent with previous studies indicating that the relationship was non-linear for both wealth with obesity and education with obesity [33, 34]. China is experiencing rapid economic development in recent years, thus results from this and previous studies may suggest that the population with high obesity in China is not those with low SES anymore [35, 36]. Considering possible reasons to obesity risk for these children, there are three plausible explanations. First, these families can afford high-calorie foods for their children due to moderate household wealth levels. Secondly, parents from these families, who hold educational level of “Middle school, High school, Technical secondary school”, may not have high health awareness to control their children’s body weight due to low education levels. Thirdly, being self-employed is usually characterized by nonstandard work schedules and may have negative impacts on parenting behaviors and child well-being [37].

According to the interaction analysis of the present study, WHR of girls in M&S families was negatively associated with neighborhood environment when compared to other SES strata, which may also serve as an explanation for the high obesity risk for children from these families. Although there has been no report of interactions between school neighborhood environment and SES, previous studies have shown interactions between home neighborhood built environment and SES on adolescents’ physical activity [10, 38]. It was reported that those living in higher-SES/higher-walkable neighborhoods had the highest moderate to vigorous physical activity (MVPA) minutes on weekends among Spanish adolescents. When only looking at low-SES neighborhoods, neighborhood walkability was positively related to MVPA among Belgian adolescents [10, 31]. It was believed that there are different interactions of built environment and SES across different countries [39]. The potential for different subgroups susceptible to the influence of the built environment in different countries is worthy of further investigation. In the present study, WHR of girls from M&S families significantly increased with increasing numbers of public transport stops and convenience stores in 1 km circular buffer of the school, and thus their disparity with other SES subgroups enlarged. However, increase in numbers of residential sites and supermarkets in 1 km circular buffer of school helped narrow the gap between M&S families and other subgroups. The results on supermarket and convenience stores are consistent with previous studies which suggested that neighborhood residents who have better access to supermarkets and limited access to convenience stores tend to have healthier diets and lower levels of obesity [40]. One Austrilia study using geographic information systems for 10,008 participants suggested that proximity to public transport stops (females) was associated with higher odds of overweight/obesity [41]. One system review provided evidence for a supportive role of residential density in promoting PA among children [42].

The present study have several implications. Firstly, for parents and teachers of children from M&S families, they should be alert to childhood obesity risk when attending schools located in areas characterized by a great many of public transport stops and convenience stores, and little supermarkets and residential sites. Secondly, for policy makers, the present study implicated that addressing the built environment may be particularly effective in narrowing the social gap compared with focusing on individual and social factors. The results suggest that a potential means to attenuate socioeconomic and gender disparities in childhood obesity risk would be for the local government to develop policies to improve the built environment in and around schools.

There were some limitations within the present study. Due to the cross-sectional design of this study, we could not infer causality of relationships. A further limitation related to the cross-sectional design was that potential self-selection bias could not be excluded. It is not clear to what extent the families in our study chose to live or study in the areas they did because the surrounding facilities were consistent with their existing lifestyle and how this might have influenced our findings. Furthermore, we included availability of food stores and transportation to characterize the neighborhood environment, but other aspects such as distance, quality, usage, and residences’ perception of the built environment have been shown in literature to play an important role in predicting weight status. This cross-sectional study provides preliminary data, but future studies including longitudinal designs and natural experiments are warranted.

## Conclusion

The present study showed that there was joint inequality of social economic status and school neighborhood environment on obesity risk among northeast Chinese girls. Those from moderate wealth & self-employed families (M&S) families may have the highest BMI and WHR. WHR of girls from M&S families changed with school neighborhood environment (availability of supermarkets, residential sites, convenience stores, and public transport stops). Local policies targeted at improving the school neighborhood environment may be one avenue for reducing socioeconomic disparities in obesity especially for girls.

## Electronic supplementary material

Below is the link to the electronic supplementary material.

Supplementary Material 1: Additional file 1

## Data Availability

The dataset(s) supporting the conclusions of this article is(are) included within the article (and its additional file(s)).
